# Supplementary material for: Amino acid variability, tradeoffs and optimality in human diet
Source: Nat Commun. 2022 Nov 5;13:6683. doi: 10.1038/s41467-022-34486-0 (PMC9637229; doi:10.1038/s41467-022-34486-0)
Supplement: Supplementary file 1 — Supplementary Information [file 41467_2022_34486_MOESM1_ESM.pdf]

Supplementary Information for

Amino acid variability, tradeoffs and optimality in human diet

Ziwei Dai[1,2], Weiyan Zheng[2], and Jason W. Locasale[1]*

1 Department of Pharmacology and Cancer Biology, Duke University School of Medicine, Durham, NC 27710, USA

2 Department of Biology, School of Life Sciences, Southern University of Science and Technology, Shenzhen 518055, China

*Corresponding author: Jason W. Locasale. Email: dr.jason.locasale@gmail.com

Supplementary Methods

1. Mathematical models of diets

1.1 Mathematical definition of diets

A diet is defined as a combination of foods consumed by an individual on a daily basis, and the consumed amount of each food included in this diet. A subset of foods (2335 foods in total) in the USDA standard reference food composition database release 28 (USDA SR28) was considered in defining diets. Other foods were discarded in this analysis due to missing values in important nutrients (i.e. carbohydrate, fat, protein, vitamins, minerals, amino acids, and other nutrients whose daily intake values are considered in the USDA 2015-2020 dietary guidelines). Thus, each diet is defined as a numeric vector with 2335 elements, each of which describes the amount of the corresponding food in this diet:

$$\boldsymbol{x} = \begin{bmatrix} x_1 \\ x_2 \\ \vdots \\ x_{2335} \end{bmatrix}$$

For instance, the variable x_1 describes the amount (in grams per day) of the first food,

which has the identifier '01001, Butter, salted' in the USDA food database, in the diet $\boldsymbol{x}$. If $x_1 = 10$, it means that an individual eating diet $\boldsymbol{x}$ consumes 10 grams of salted butter per day. Since one cannot consume negative amount of foods, all elements of $\boldsymbol{x}$ are nonnegative, i.e. $x_i \geq 0, i = 1,2,\cdots,2335$.

1.2 Nutritional values of foods and diets

The nutritional profile of a food is defined by the amount of each nutrient in one gram of the given food. Thus, for each nutrient, there are 2335 values that describe the levels of this nutrient in the 2335 foods. Hence, for each nutrient, we define a 2335-dimensional nutrient abundance vector $\boldsymbol{b}$ that quantifies the abundances of this nutrient in the 2335 foods:

$$\boldsymbol{b} = \begin{bmatrix} b_1 \\ b_2 \\ \vdots \\ b_{2335} \end{bmatrix}$$

The element b_i describes the amount of the corresponding nutrient in one gram of the *i*-th food. For instance, considering the nutrient 'Total lipid (fat)', b_1 has the value 0.8111, meaning that each gram of the first food, which has the identifier '01001, Butter, salted', contains 0.8111 gram of fat in total.

Based on the definitions of the diet vector $\boldsymbol{x}$ and nutrient abundance vector $\boldsymbol{b}$ for a nutrient, the total amount of this nutrient in the diet $\boldsymbol{x}$ is $r = \boldsymbol{b}^T\boldsymbol{x}$. Hence, the total amount of a nutrient in a diet is a linear function of amounts of foods in this diet with linear coefficients determined by abundances of this nutrient in different foods.

We next consider the abundances of *m* different nutrients at the same time. For the *i*-th nutrient, let $\boldsymbol{b}_i$ denote the nutrient abundance vector for this nutrient, we can then compute the amounts of nutrients $1,2,\cdots,m$ in the diet $\boldsymbol{x}$:

$$r_i = \boldsymbol{b}_i^T\boldsymbol{x}$$

Thus, the nutritional composition $\boldsymbol{r}$ of the diet $\boldsymbol{x}$ can be written in the following form:

$$\boldsymbol{r} = \begin{bmatrix} r_1 \\ r_2 \\ \vdots \\ r_m \end{bmatrix} = \begin{bmatrix} \boldsymbol{b}_1^T \boldsymbol{x} \\ \boldsymbol{b}_2^T \boldsymbol{x} \\ \vdots \\ \boldsymbol{b}_m^T \boldsymbol{x} \end{bmatrix} = [\boldsymbol{b}_1 \quad \boldsymbol{b}_2 \quad \cdots \quad \boldsymbol{b}_m]^T \, \boldsymbol{x}$$

Let $\mathbf{B} = [\boldsymbol{b}_1 \quad \boldsymbol{b}_2 \quad \cdots \quad \boldsymbol{b}_m]^T$, then we have $\boldsymbol{r} = \mathbf{B}\boldsymbol{x}$. The element B_{ij} in the matrix $\mathbf{B}$ denotes the abundance of the i-th nutrient in the j-th food.

Specifically, we quantify the abundance of amino acids in foods and diets using similar notation. Let $\mathbf{A} = [\boldsymbol{a}_1 \quad \boldsymbol{a}_2 \quad \cdots \quad \boldsymbol{a}_{18}]^T$ denote the abundances of amino acids in the 2335 foods (i.e. columns of $\mathbf{A}$ are nutrient abundance vectors for amino acids), we can compute the amino acid composition $\boldsymbol{s}$ of each diet $\boldsymbol{x}$ by $\boldsymbol{s} = \mathbf{A}\boldsymbol{x}$. The reason that 18 instead of 20 amino acid variables are considered is that the current protocol for quantification of amino acids in foods requires hydrolysis of protein, which breaks protein-bound amino acids into free amino acids, before quantification of the amino acids. During the protein hydrolysis, the amides glutamine and asparagine are converted to glutamic acid and aspartic acid, respectively.

1.3 Mathematical definition of human dietary patterns

Human dietary patterns are representative modes of diets consumed in certain geographical regions, consumed by certain ethnic groups, or recommended by certain dietary guidelines for the goal of improving health. Examples of human dietary patterns include Mediterranean diet, Japanese diet, Paleo diet and plant-based diet, which are defined by consumption of certain combinations of foods, or ketogenic diet and Atkins diet, which are defined by limited intake of certain nutrients. Thus, each human dietary pattern can be defined mathematically as a set of constraints on the composition of foods or nutrients in a diet that falls into this category of dietary pattern.

1.3.1 Constraints on foods

This type of constraint limits the total amount of certain food allowed in a diet. These constraints set either an upper bound or a lower bound of daily consumption of the corresponding food or

group of foods.

For example, in a plant-based diet, consumption of animal products, such as meat, seafood, egg and dairy products, is strictly prohibited. Thus, for each food, we can define a binary label indicating whether this food is animal-based or not:

$$d_i = \begin{cases} 1, & \text{if the i-th food is animal based} \\ 0, & \text{otherwise} \end{cases}$$

A diet is a plant-based diet if and only if the total amount of animal-based foods in this diet equals zero, i.e. $\boldsymbol{d}^T\boldsymbol{x} = 0$. In other words, every diet that satisfies this linear constraint is a plant-based diet, hence the linear constraint $\boldsymbol{d}^T\boldsymbol{x} = 0$ gives a definition of plant-based diet.

Another example is the Mediterranean diet, in which 3 to 6 servings of cereals are consumed each day. Similar to the case of plant-based diet, we can define a vector $\boldsymbol{d}$:

$$\boldsymbol{d} = \begin{bmatrix} d_1 \\ d_2 \\ \vdots \\ d_{2335} \end{bmatrix}$$

$$d_i = \begin{cases} \frac{1}{w_i}, & \text{if the i-th food is a cereal-based food} \\ 0, & \text{otherwise} \end{cases}$$

In which w_i is the weight of one serving of the *i*-th food. Thus, in a Mediterranean diet, the daily intake of 3 to 6 servings of cereals can be represented by the linear constraint:

$$3 \leq \boldsymbol{d}^T\boldsymbol{x} \leq 6$$

Similar constraints on daily consumption of other groups of foods, such as seafood, fruits, legumes and so on, can also be defined in this way. These constraints together offer a definition of Mediterranean diet (see Fig S2 for a complete list of food groups included in Mediterranean diet). A diet is a Mediterranean diet if and only if it satisfies all of these constraints on allowed numbers of servings of foods.

A general form of this type of constraint can be written as below:

$$\boldsymbol{l}_f \leq \mathbf{D}\boldsymbol{x} \leq \boldsymbol{u}_f$$

In which $\boldsymbol{l}_f$ and $\boldsymbol{u}_f$ are the lower and upper bounds of the total amount of foods in each category whose total daily consumption is constrained in a specific range in this diet.

1.3.2 Constraints on absolute levels of nutrient intake

This type of constraint limits the total daily intake of a nutrient in a diet. Similar to the constraints on the amount of foods, these constraints also determine upper or lower bounds of nutrient intake. For instance, in the Atkins diet, which is a carbohydrate-restricted diet, the recommended total daily intake of carbohydrate is no more than 20 grams. As we have discussed in section **1.2**, the total daily intake of carbohydrate in a diet $\boldsymbol{x}$ is $\boldsymbol{c}^T\boldsymbol{x}$, in which $\boldsymbol{c}$ is the vector of carbohydrate contents of foods. Thus, the Atkins diet can be defined by the linear constraint:

$$\boldsymbol{c}^T\boldsymbol{x} \leq 20$$

This type of constraints can be written in the general form below:

$$\boldsymbol{l}_n \leq \mathbf{C}\boldsymbol{x} \leq \boldsymbol{u}_n$$

In which $\boldsymbol{l}_n$ and $\boldsymbol{u}_n$ are the lower and upper bounds of the total daily intake of nutrients related to this diet, and the *i*-th row in the matrix $\mathbf{C}$ is the nutrient abundance vector of the *i*-th of these nutrients.

1.3.3 Constraints on ratios of nutrient intake

This type of constraint determines allowed ranges of ratios between two nutrients. Most of these constraints are about the percentage contribution of a macronutrient, such as carbohydrate, to the total daily intake of calories. For instance, in a ketogenic diet, at least 70% of the total daily calories come from fat, while a very small fraction (e.g. less than 5%) comes from carbohydrate. Let $\boldsymbol{c}$, $\boldsymbol{f}$ and $\boldsymbol{e}$ denote the nutrient abundance vectors for carbohydrate, fat and calories, these constraints on the relationship between intake of fat, carbohydrate and calories in a ketogenic diet can be written as below:

$$\begin{cases} \boldsymbol{c}^T\boldsymbol{x} \leq 0.05\boldsymbol{e}^T\boldsymbol{x} \\ \boldsymbol{f}^T\boldsymbol{x} \geq 0.7\boldsymbol{e}^T\boldsymbol{x} \end{cases}$$

Rearranging the terms at the two ends of the inequalities, we have:

$$[\boldsymbol{c} - 0.05\boldsymbol{e} \quad 0.7\boldsymbol{e} - \boldsymbol{f}]^T\boldsymbol{x} \leq \mathbf{0}$$

Thus, a general form of this type of constraints is:

$$\mathbf{E}\boldsymbol{x} \le \mathbf{0}$$

1.3.4 The general mathematical form for definition of a diet

Summarizing the types of constraints in sections **1.3.1**, **1.3.2** and **1.3.3**, we have the general form of the definition of a diet:

$$\begin{cases} \boldsymbol{x} \ge \mathbf{0} \\ \boldsymbol{l}_n \le \mathbf{C}\boldsymbol{x} \le \boldsymbol{u}_n \\ \boldsymbol{l}_f \le \mathbf{D}\boldsymbol{x} \le \boldsymbol{u}_f \\ \mathbf{E}\boldsymbol{x} \le \mathbf{0} \end{cases}$$

It is worth noting that all of these constraints are linear.

2. Quantification of amino acid levels in diets

The goal of this part of analysis is to quantify abundance of amino acids in human diets and identify quantitative amino acid signatures for each dietary pattern such as Mediterranean, American diet and ketogenic diet. For each amino acid, two metrics are used to quantify their enrichment in a diet: one is the absolute daily intake of this amino acid in a diet, the other one is the fraction of this amino acid in the total daily intake of all amino acids. As we have discussed previously, there are 18 variables describing absolute levels of amino acids in foods and diets. These variables are the amounts of serine, tyrosine, glycine, phenylalanine, proline, valine, lysine, leucine, isoleucine, tryptophan, arginine, methionine, histidine, threonine, alanine, cystine, aspartate + asparagine, and glutamate + glutamine. The units are gram amino acid per gram weight of food (for amino acid levels in foods) and gram amino acid per day (for amino acid levels in diets).

2.1 Calculation of ranges of amino acid levels in diets

As we have discussed in section 1.2, the absolute levels of amino acids in a diet $\boldsymbol{x}$ are $\boldsymbol{s} = \mathbf{A}\boldsymbol{x}$, and the absolute level of the *i*-th amino acid in this diet is $s_i = \boldsymbol{a}_i^T\boldsymbol{x}$. Hence, with the mathematical definition of a dietary pattern, which we have developed in the previous section, the lowest allowed intake of the *i*-th amino acid in this dietary pattern can be computed by

solving the linear programming problem:

$$\min \boldsymbol{a}_i^T \boldsymbol{x}, \text{ s.t.}$$

$$\begin{cases} \boldsymbol{x} \geq \boldsymbol{0} \\ \boldsymbol{l}_n \leq \mathbf{C}\boldsymbol{x} \leq \boldsymbol{u}_n \\ \boldsymbol{l}_f \leq \mathbf{D}\boldsymbol{x} \leq \boldsymbol{u}_f \\ \mathbf{E}\boldsymbol{x} \leq \boldsymbol{0} \end{cases}$$

And the highest allowed intake of the i-th amino acid in this dietary pattern can be computed by solving another linear programming problem:

$$\max \boldsymbol{a}_i^T \boldsymbol{x}, \text{ s.t.}$$

$$\begin{cases} \boldsymbol{x} \geq \boldsymbol{0} \\ \boldsymbol{l}_n \leq \mathbf{C}\boldsymbol{x} \leq \boldsymbol{u}_n \\ \boldsymbol{l}_f \leq \mathbf{D}\boldsymbol{x} \leq \boldsymbol{u}_f \\ \mathbf{E}\boldsymbol{x} \leq \boldsymbol{0} \end{cases}$$

2.2 Sampling of amino acid composition in diets

Amino acid composition of a diet, or relative levels of amino acids in a diet, is defined as a vector $\boldsymbol{r}$ that contains ratios of absolute levels of amino acids to total amount of all amino acids in this diet:

$$r_i = \frac{s_i}{\sum_{j=1}^{18} s_j} = \frac{\boldsymbol{a}_i^T \boldsymbol{x}}{\sum_{j=1}^{18} \boldsymbol{a}_j^T \boldsymbol{x}} = \frac{\boldsymbol{a}_i^T \boldsymbol{x}}{[1 \quad \cdots \quad 1] \cdot \mathbf{A}\boldsymbol{x}}$$

Since this is no longer a linear function of $\boldsymbol{x}$, ranges of r_i cannot be computed using linear programming. Instead, we first uniformly sampled absolute levels of amino acids in all diets consistent with this dietary pattern (i.e. diets that satisfy all linear constraints set by definition of this dietary pattern):

$$\text{Uniformly sample } \boldsymbol{s} = \mathbf{A}\boldsymbol{x}, \text{ s.t.}$$

$$\begin{cases} \boldsymbol{x} \geq \boldsymbol{0} \\ \boldsymbol{l}_n \leq \mathbf{C}\boldsymbol{x} \leq \boldsymbol{u}_n \\ \boldsymbol{l}_f \leq \mathbf{D}\boldsymbol{x} \leq \boldsymbol{u}_f \\ \mathbf{E}\boldsymbol{x} \leq \boldsymbol{0} \end{cases}$$

Because all constraints on $\boldsymbol{x}$ are linear constraints, the feasible region for $\boldsymbol{x}$, i.e. the region in which a diet $\boldsymbol{x}$ falls in if and only if $\boldsymbol{x}$ is a diet consistent with this certain dietary pattern, is

a polyhedron. Thus, because a polyhedron after a linear transformation is still a polyhedron, the feasible region for $\mathbf{A}\boldsymbol{x}$ is also a polyhedron. Let Ω denote this polyhedron. It was then uniformly sampled using the hit-and-run sampling algorithm with a direction choice strategy for accelerated convergence (ACHR, Supplementary Figure 11)[1], generating *N* samples $\boldsymbol{s}_1, \boldsymbol{s}_2, \cdots, \boldsymbol{s}_N$. These samples were then transformed to relative ratios of amino acids by the dividing total amount of amino acids in each sample:

$$\boldsymbol{r}_n = \frac{\boldsymbol{s}_n}{\sum_{i=1}^{18} s_{ni}}, n = 1,2, \cdots, N$$

In each iteration of the ACHR algorithm, a direction for perturbation, $\boldsymbol{d}$, is selected using different strategies based on the current phase of sampling (i.e. warm-up phase or main phase, Supplementary Figure 11a), and the new sample $\boldsymbol{s}_{i+1}$ is generated by randomly perturb the current sample $\boldsymbol{s}_i$ on the direction $\boldsymbol{d}$ by the step size k (Supplementary Figure 11a):

$$\boldsymbol{s}_{i+1} = \boldsymbol{s}_i + k \cdot \boldsymbol{d}$$

Since the new sample $\boldsymbol{s}_{i+1}$ still needs to be in the feasible region, we have:

$$\begin{cases} \boldsymbol{s}_i + k \cdot \boldsymbol{d} = \mathbf{A}\boldsymbol{x} \\ \boldsymbol{x} \geq \mathbf{0} \\ \boldsymbol{l}_n \leq \mathbf{C}\boldsymbol{x} \leq \boldsymbol{u}_n \\ \boldsymbol{l}_f \leq \mathbf{D}\boldsymbol{x} \leq \boldsymbol{u}_f \\ \mathbf{E}\boldsymbol{x} \leq \mathbf{0} \end{cases}$$

Thus, the minimal and maximal allowed values for k can be determined by solving the linear programming problems:

$$\text{min(or max) } k, \text{ s.t.}$$

$$\begin{cases} [\mathbf{A} \quad -\boldsymbol{d}] \begin{bmatrix} \boldsymbol{x} \\ k \end{bmatrix} = \boldsymbol{s}_i \\ \boldsymbol{x} \geq \mathbf{0} \\ \boldsymbol{l}_n \leq \mathbf{C}\boldsymbol{x} \leq \boldsymbol{u}_n \\ \boldsymbol{l}_f \leq \mathbf{D}\boldsymbol{x} \leq \boldsymbol{u}_f \\ \mathbf{E}\boldsymbol{x} \leq \mathbf{0} \end{cases}$$

Let $[a, b]$ denote the range of k determined by solving these two linear programming problems, we then randomly choose the value of k from the uniform distribution on $[a, b]$ and generate the new sample $\boldsymbol{s}_{i+1}$ based on the randomly selected k:

$$k \sim U(a, b)$$

$$s_{i+1} = s_i + k \cdot d$$

To determine the number N of samples sufficient to thoroughly capture the distribution of amino acid compositions in a dietary pattern, we performed the ACHR sampling using different sample sizes for the USDA-recommended diet and examined the trends of mean and standard deviation against number of samples. We used the USDA-recommended diet here to determine N because the definition of this dietary pattern includes the largest number of constraints among all dietary patterns studied here, thus likely exhibiting higher complexity in the sampling process compared to other dietary patterns. We found that for all amino acids, the mean and standard deviation become largely unchanged by increasing N when N is larger than 40,000. Thus, we used $N = 50{,}000$ to sample all dietary patterns. All linear programming problems were solved using the function 'mosekopt' in the MOSEK Optimization Toolbox for MATLAB[2].

3. Reconstruction of amino acid profiles in human diets

3.1 Acquisition of datasets

Microsoft Access database files for USDA National Nutrient Database for Standard Reference (SR) and the USDA Food and Nutrient Database for Dietary Studies (FNDDS) were downloaded from the website for USDA Agricultural Research Service: https://data.nal.usda.gov/dataset/usda-national-nutrient-database-standard-reference-legacy-release (SR), and https://www.ars.usda.gov/northeast-area/beltsville-md-bhnrc/beltsville-human-nutrition-research-center/food-surveys-research-group/docs/fndds-download-databases/ (FNDDS). Matching between versions of the SR and FNDDS databases is shown in Supplementary Table 1:

Supplementary Table 1. Matching between versions of FNDDS and SR databases

Year	FNDDS database version	SR database version
**2007-2008**	FNDDS 2007-2008	Release 22
**2009-2010**	FNDDS 2009-2010	Release 24

**2011-2012**	FNDDS 2011-2012	Release 26
**2013-2014**	FNDDS 2013-2014	Release 28

Nutritional composition values of foods in the SR databases were retrieved from the table 'NUT_DATA' in the corresponding MS Access database files for the SR databases. Mappings from foods in the SR databases to foods in the FNDDS databases were retrieved from the table 'FNDDSSRLinks' in the MS Access database files for the FNDDS databases. Factors for moisture and fat adjustments (i.e. factors quantifying the amount of water or fat lost or gained during the cooking or food preparation processes for some foods in the database) were retrieved from the table 'MoistNFatAdjust' in the FNDDS database files. Retention factors for nutrients (i.e. factors quantifying the fraction of nutrients kept during cooking process) were obtained from the USDA Ag Data Commons website: https://data.nal.usda.gov/dataset/usda-table-nutrient-retention-factors-release-6-2007.

SAS (.xpt) files for NHANES 2007-2008, 2009-2010, 2011-2012, 2013-2014, 2015-2016 datasets, including demographics data, dietary data, examination data, laboratory data and questionnaire data were retrieved from the web page for NHANES: https://wwwn.cdc.gov/nchs/nhanes/Default.aspx, and converted to R data frames using the function 'sasxport.get()' in the R package 'Hmisc'.

3.2 Comparison of data imputation methods

Two methods for missing data imputation, random forest (RF) and predictive mean matching (PMM), were applied to the USDA SR Release 28 dataset to evaluate their performance. RF-based imputation was performed using the function 'missForest()' in the R package 'missForest'[3]. PMM-based imputation was performed using the function 'mice()' in the R package 'MICE'[4]. For each nutrient variable, we first calculated a missing ratio defined by the number of missing values for this variable divided by the total number of foods (i.e. 8788) in the dataset. All nutrient variables with missing ratio higher than 0.6 were discarded before the

following analysis. We then adopted two alternative strategies for data transformation before the imputation. In one strategy (without transformation), the raw abundances of nutrients, including amino acids, were used as the input to the data imputation algorithms. In the other strategy, absolute abundances of amino acids were transformed to the ratio of absolute amino acid levels to one plus the protein level:

$$\widehat{Y_{\mathrm{AA}}} = \frac{Y_{\mathrm{AA}}}{1 + Y_{\mathrm{protein}}}$$

The transformed amino acid variables were then used together with raw values of other nutrient variables as the input to the data imputation algorithms. The major goal of performing this transformation is to decouple the variation in relative levels of amino acids and variation in absolute abundances of protein. After imputation has been done for the transformed variables $\widehat{Y_{\mathrm{AA}}}$, imputation for absolute levels of amino acids can be calculated from the imputed $\widehat{Y_{\mathrm{AA}}}$ values:

$$Y_{\mathrm{AA}}^{(\mathrm{i})} = \widehat{Y_{\mathrm{AA}}^{(\mathrm{i})}}\left(1 + Y_{\mathrm{protein}}^{(\mathrm{i})}\right)$$

In which the superscript (i) indicates imputed variables.

Combining the two imputation algorithms (i.e. RF and PMM) and two data transformation strategies, four data imputation scenarios were considered in the comparison. These include RF without data transformation, RF with data transformation, PMM without data transformation, and PMM with data transformation. Performance of these data imputation scenarios were evaluated using 5-fold cross validation, in which all values (including known values and missing values) in the input dataset were split into five groups. In the *n*-th (n=1,2,3,4,5) iteration, values in the *n*-th group were treated as the missing values which were then imputed using data points in other groups. After all five iterations were completed, the actual values and imputed values were compared for each nutrient using Spearman's rank correlation coefficient. Distributions of these Spearman's rank correlation coefficients were then compared across data imputation scenarios to determine the strategy with best performance (i.e. data imputation scenario that reached the highest Spearman correlation between the imputed and actual values). Spearman correlation coefficients between actual and imputed values for pairwise ratios between amino

acid abundances (e.g. ratio of serine level to glycine level, etc) were also computed to evaluate the abilities of the data imputation methods to correctly capture relative abundances of amino acids in addition to their absolute levels. Since RF with data transformation showed the highest Spearman correlation coefficients between the actual and imputed values for both single nutrients (Supplementary Figure 4b) and pairwise ratios for amino acid abundances (Supplementary Figure 4c), this strategy was selected for imputation of all USDA SR datasets and FNDDS datasets.

3.3 Imputation of USDA SR and FNDDS datasets

Missing data imputation was first done for nutritional composition data of foods in the USDA SR datasets. Nutrients with missing values in more than 60% of the foods were removed from the datasets before imputation. Amino acid abundances were transformed by dividing one plus the protein abundance as described in the previous section. Imputation was performed using the random forest regression method implemented in the function 'missForest()' in the R package 'missForest' with default parameters. Nutritional composition values of foods in the FNDDS datasets were computed using the imputed USDA SR datasets, the mapping information from foods in SR to foods in FNDDS, factors for moisture and fat adjustments, and retention factors for nutrients.

A typical food in the FNDDS dataset is prepared from one or more foods in the USDA SR dataset with the proportion for each food is provided together with its identifier in USDA SR. For instance, the FNDDS food with identifier '27520130' and description 'Bacon, chicken, and tomato club sandwich, with lettuce and spread' is mapped to six foods in the USDA SR database:

Supplementary Table 2. Mapping between the USDA SR database and the food 'Bacon, chicken and tomato club sandwich' in the USDA FNDDS dataset

USDA SR ID	Food name	Weight (g)
**18069**	Bread, white, commercially prepared (includes soft bread crumbs)	78

**5064**	Chicken, broilers or fryers, breast, meat only, cooked, roasted	85
**10124**	Pork, cured, bacon, cooked, broiled, pan-fried or roasted	19
**11252**	Lettuce, iceberg (includes crisphead types), raw	25
**11529**	Tomatoes, red, ripe, raw, year round average	30
**4025**	Salad dressing, mayonnaise, soybean oil, with salt	13.75

Assuming that there are *m* foods in the USDA FNDDS database and *n* foods in the USDA SR database, we can define a $m \times n$ matrix $\mathbf{M}$ to describe the mapping from USDA SR to USDA FNDDS, in which the element M_{ij} describes the amount of the *j*-th food in the USDA SR database needed in the preparation of the *i*-th food in the USDA FNDDS database. Let $\mathbf{Y}$ denote the nutritional composition of foods (i.e. amounts of nutrients in 1 gram of each food) in the USDA SR database, we can then compute the nutritional composition of the *i*-th food in the USDA FNDDS database:

$$\boldsymbol{z}_i = \frac{\sum_{j=1}^{n} M_{ij}\mathbf{R}_{ij}\boldsymbol{y}_j}{\sum_{j=1}^{n} M_{ij}}$$

in which $\mathbf{R}_{ij}$ is a diagonal matrix with the *k*-th diagonal element being the retention factor for the *k*-th nutrient in preparation of the *i*-th FNDDS food from the *j*-th SR food. A retention factor of 0.1 means that 10% of this nutrient is preserved during the food preparation process while the rest 90% is lost. This nutritional composition vector $\boldsymbol{z}_i$ was then further corrected for moisture and fat adjustments:

$$z_{ij}^{\text{(adjusted)}} = \begin{cases} \dfrac{100 \times z_{ij}}{100 + a^{(m)} + \sum_{k=1}^{n_f} a_k^{(f)}} & \text{if the } j\text{-th nutrient is neither water nor fat} \\ \dfrac{100 \times z_{ij} + a^{(m)}}{100 + a^{(m)} + \sum_{k=1}^{n_f} a_k^{(f)}} & \text{if the } j\text{-th nutrient is water} \\ \dfrac{100 \times z_{ij} + a_k^{(f)}}{100 + a^{(m)} + \sum_{k=1}^{n_f} a_k^{(f)}} & \text{if the } j\text{-th nutrient is the } k\text{-th fat adjusted} \end{cases}$$

in which $a^{(m)}$ is the moisture adjustment factor which quantifies grams of water gained or lost during cooking of 100 grams of raw food, n_f is the total number of different types of fat,

$\sum_{k=1}^{n_f} a_k^{(f)}$ is the total fat adjustment factor that quantifies total grams of fat gained or lost during cooking of 100 grams of raw food, $a_k^{(f)}$ is the fat adjustment factor that quantifies grams of the k-th type of fat (e.g. polyunsaturated fat, saturated fat 16:0, etc) gained or lost during cooking of 100 grams of raw food. The adjusted nutritional values for foods in FNDDS, including amino acid abundances in all FNDDS foods which were not included in the original FNDDS datasets, were used in the following reconstruction of amino acid intake profiles in the NHANES dietary records.

3.4 Reconstruction of amino acid intake profiles in NHANES dietary recalls

Nutrient composition values for dietary recalls in NHANES 2007-2008, 2009-2010, 2011-2012 and 2013-2014 datasets were computed based on the reconstructed nutritional values for foods in FNDDS and food consumption records in human subjects in the NHANES datasets (i.e. data files with the description 'Dietary Interview – Individual Foods, First Day/Second Day') retrieved from the web pages of NHANES. For each human subject, the food consumption record on one day (day 1 or day 2) includes a group of foods in FNDDS and grams of each food consumed on that day. An example of a food consumption record is shown in Supplementary Table 3 below:

Supplementary Table 3. An example of food consumption record in NHANES

**seqn**<br>(Identifier of human subject)	**dr1ifdcd**<br>(FNDDS food ID)	**dr1igrms**<br>(Grams of food)
41475	64132010	160
41475	92101500	710.4
41475	91200040	4
41475	12120100	10.08
41475	52215200	63.62
41475	22600100	10

41475	71000100	20.13
41475	72201200	30
41475	75221000	4.48
41475	32104900	122
41475	94000100	474
41475	92302300	1554
41475	94000100	474
41475	27214110	134.3
41475	58145110	182.25
41475	75216123	42.25
41475	92302300	473.6
41475	53410100	122.06
41475	13110100	16.63
41475	94000100	59.25

Let $\mathbf{Z}$ denote the reconstructed nutritional values (which include the reconstructed amino acid abundances in these foods) for foods in FNDDS and $\boldsymbol{x}$ denote a food consumption vector in which the i-th value quantifies the total grams of the i-th food consumed on that day, we can compute the nutrient intake profile, which includes the uptake of amino acids, in this dietary record:

$$\boldsymbol{y} = \frac{\mathbf{Z} \cdot \boldsymbol{x}}{\|\boldsymbol{x}\|_1}$$

in which $\|\boldsymbol{x}\|_1$ is the L1-norm of the food consumption vector (i.e. total weight of food consumed on that day).

3.5 Comparison of model-predicted and actual amino acid signatures of ketogenic diet

To quantify the amino acid signature of ketogenic diet according to the NHANES dietary data, for each dietary intake profile of an individual in the NHANES dataset, a ketogenic score quantifying this person's adherence to the ketogenic diet was defined as below:

$$K = -(\max(f_c - 0.05, 0))^2 - (\min(f_l - 0.7, 0))^2$$

In which f_c is the fraction of calories from dietary intake carbohydrate, and f_l is the fraction of calories from dietary intake of fat. For the *i*-th amino acid, we then computed the Spearman's rank correlation coefficient between its intake and the ketogenic score:

$$c_i = \rho(\boldsymbol{K}, \boldsymbol{A}_i)$$

In which $\boldsymbol{K}$ is the vector storing the ketogenic score of all individuals in the NHANES data, $\boldsymbol{A}_i$ is the vector storing the intake of the i-th amino acid of all individuals in the NHANES data, ρ is the Spearman's rank correlation coefficient. The computed correlation coefficients indicate associations between dietary intake of amino acids and adherence to ketogenic diet: amino acids enriched in ketogenic diets will have positive correlation coefficients, while amino acids with lower intake in ketogenic diet will have negative correlation coefficients. Hence, they were able to serve as indicators of amino acid signatures associated with ketogenic diet. This amino acid signature of ketogenic diet in NHANES data was then compared with the amino acid profile of ketogenic diet predicted by our modeling framework using linear programming (defined as the difference between mean amino acid abundance in computationally sampled ketogenic versus other diets).

4. Analysis of association between dietary amino acids and human health

4.1 Definition of human health variables

Binary disease variables indicating the presence of pathological conditions, including obesity, hypertension, diabetes, and cancer, were constructed based on the datasets 'Examination data', 'Laboratory data', and 'Questionnaire data' in the NHANES databases 2007-2008, 2009-2010, 2011-2012, and 2013-2014. Adults with BMI values higher than 30 were considered obese. Hypertension was defined as the condition of systolic blood pressure (the variables 'bpxsy1', 'bpxsy2' and 'bpxsy3', corresponding to three consecutive measurements) being higher than 120 mm Hg and diastolic blood pressure (the variables 'bpxdi1', 'bpxdi2' and 'bpxdi3', corresponding to three consecutive measurements) being higher than 80 mm Hg. Diabetes was

defined as the condition of glycohemoglobin levels (the variable 'lbxgh') being higher than 6.5%, fasting plasma glucose concentration (the variable 'lbxglu') higher than 126 mg/dL, and blood glucose concentration in response to oral glucose tolerance test (the variable 'lbxglt') higher than 200 mg/dL. Information about the presence of cancer was obtained from answers to the question 'Have you ever been told by a doctor or other health professional that you had cancer or a malignancy of any kind?' in the questionnaire about medical conditions, in which the answers 'yes' or 'no' were linked to the presence or absence of cancer, while the answers 'refused' and 'don't know' were considered as missing data.

4.2 Correlation analysis

Partial Spearman correlation coefficients between dietary amino acid composition or other nutritional variables and health variables defined in the previous section were computed using the MATLAB function 'partialcorr', controlling for demographic and lifestyle-related factors including income, education, age, gender, ethnicity, marital status, smoking, alcohol consumption, physical activity, and batch. Only adults (i.e. age > 20 years old) were included in the analysis. Individuals with dietary intake of any nutrient higher than three times of the 99th percentile of the intake of that nutrient among the population were considered outliers and not included in the following analysis.

4.3 Machine learning

A logistic regression model with elastic net regularization of the regression coefficients was built to predict disease prevalence from dietary variables. Under the assumption that the interactions between dietary variables and potential confounders are additive, the model has the form below:

$$p(y=1|\boldsymbol{x}_{AA},\boldsymbol{x}_{nut},\boldsymbol{x}_c)=\frac{e^{(\boldsymbol{w}_{AA}{}^T\boldsymbol{x}_{AA}+\boldsymbol{w}_{nut}{}^T\boldsymbol{x}_{nut}+\boldsymbol{w}_c{}^T\boldsymbol{x}_c+b)}}{1+e^{(\boldsymbol{w}_{AA}{}^T\boldsymbol{x}_{AA}+\boldsymbol{w}_{nut}{}^T\boldsymbol{x}_{nut}+\boldsymbol{w}_c{}^T\boldsymbol{x}_c+b)}}$$

This model links dietary amino acid composition ($\boldsymbol{x}_{AA}$), other dietary variables ($\boldsymbol{x}_{nut}$), and potential confounders ($\boldsymbol{x}_c$) to the disease outcome (y, value 1 means that the individual has that disease). The R package "glmnet" was used to train the model and assess its performance using 5-fold cross validation with the survey weight of each sample in the NHANES dataset taken into

consideration. Feature importance for each variable in x_{AA}, x_{nut} and x_c was computed by absolute value of the standardized regression coefficient (i.e. the product of the original regression coefficient and the standard deviation of the variable). Variables with non-zero regression coefficients were considered as variables affecting the disease outcomes. Nutritional variables were grouped into seven categories: energy, vitamins, minerals, macronutrients, macronutrient compositions, amino acid compositions, other nutrients. The complete table of nutritional variables and the groups they belong to is included below (Supplementary Table 4). Fraction of variables that affect the disease outcome in each group was computed by dividing the number of variables with non-zero regression coefficient by the total number of variables in that group.

Supplementary Table 4. Seven categories of nutritional variables

**Energy**	**Macronutrients**	**Macronutrient subtypes**	**Vitamins**	**Minerals**	**Amino acids**	**Others**
Energy(kcal)	Carbohydrate(gm)	PFA 18:2(Octadecadienoic)(gm)	Food folate(mcg)	Zinc(mg)	Serine	Caffeine(mg)
	Total fat(gm)	Total monounsaturated fatty acids(gm)	Vitamin K(mcg)	Iron(mg)	Tyrosine	Theobromine(mg)
	Protein(gm)	PFA 18:3(Octadecatrienoic)(gm)	Niacin(mg)	Copper(mg)	Glycine	
		SFA 16:0(Hexadecanoic)(gm)	Riboflavin (Vitamin B2)(mg)	Phosphorus(mg)	Phenylalanine	
		Dietary fiber(gm)	Folate,DFE(mcg)	Calcium(mg)	Proline	

		Total sugars(gm)	Total choline(mg)	Sodium(mg)	Valine	
		Total polyunsaturated fatty acids(gm)	Vitamin C(mg)	Selenium(mcg)	Lysine	
		MFA 18:1 (Octadecenoic)(gm)	Vitamin A,RAE(mcg)	Magnesium(mg)	Aspartate+Asparagine	
		SFA 18:0(Octadecanoic)(gm)	Vitamin B12(mcg)	Potassium(mg)	Leucine	
		Total saturated fatty acids(gm)	Vitamin E as alpha-tocopherol(mg)		Isoleucine	
			Thiamin(Vitamin B1)(mg)		Tryptophan	
			Vitamin B6(mg)		Arginine	
			Vitamin D(D2+D3)(mcg)		Glutamate+Glutamine	
			Folic acid(mcg)		Methionine	
					Histidine	
					Threonine	
					Alanine	
					Cystine	

5 Development of the diet designer

5.1 Identification of amino acids associated with obesity

For each amino acid, the regression coefficient of that amino acid in the logistic regression model linking the dietary variables to obesity prevalence and the partial Spearman's rank correlation coefficient between this amino acid and obesity prevalence were used in combination to determine whether dietary intake of this amino acid is associated with obesity. An amino acid with both positive partial Spearman's rank correlation coefficient and positive regression coefficient was identified as positively associated with obesity, while an amino acid with both negative partial Spearman's rank correlation coefficient and negative regression coefficient was considered negatively associated with obesity. Otherwise, the amino acid was considered not associated with obesity. Similar rules were also used to identify amino acids positively associated with diabetes and amino acids negatively associated with diabetes. Computation of the regression coefficients and partial Spearman's rank correlation coefficients was performed based on the NHANES 2007-2014 datasets.

5.2 Analysis of Pareto optimality

Based on the amino acids positively or negatively associated with obesity incidence that were found in the previous section, we defined two objectives for optimization of dietary amino acid intake, that is, to maximize the total intake of amino acids negatively associated with obesity incidence (i.e. AAs-to-maximize), and to minimize the total intake of amino acids positively associated with obesity incidence (i.e. AAs-to-minimize). Let $\boldsymbol{a}_{+}$ and $\boldsymbol{a}_{-}$ denote the vectors consisting of total abundance of AAs-to-maximize and AAs-to-minimize in each food, then the inner products $\boldsymbol{a}_{+}^{T}\boldsymbol{x}$ and $\boldsymbol{a}_{-}^{T}\boldsymbol{x}$ indicate the total intake of AAs-to-maximize and AAs-to-minimize in a diet $\boldsymbol{x}$. Therefore, we have the mathematical form of optimizing the two amino acid intake goals for a specific dietary pattern with the general form described in section 1.3.4:

$$\max \boldsymbol{a}_{+}^{T}\boldsymbol{x}, \min \boldsymbol{a}_{-}^{T}\boldsymbol{x}, \text{s.t.}$$

$$\begin{cases} \boldsymbol{x} \geq \mathbf{0} \\ \boldsymbol{l}_n \leq \mathbf{C}\boldsymbol{x} \leq \boldsymbol{u}_n \\ \boldsymbol{l}_f \leq \mathbf{D}\boldsymbol{x} \leq \boldsymbol{u}_f \\ \mathbf{E}\boldsymbol{x} \leq \mathbf{0} \end{cases}$$

A feasible solution $\boldsymbol{x}_0$ of this problem is defined as a Pareto solution if for any other feasible solution $\boldsymbol{x}_1$, $\boldsymbol{a}_{-}^{T}\boldsymbol{x}_1 > \boldsymbol{a}_{-}^{T}\boldsymbol{x}_0$ if $\boldsymbol{a}_{+}^{T}\boldsymbol{x}_1 > \boldsymbol{a}_{+}^{T}\boldsymbol{x}_0$, and $\boldsymbol{a}_{+}^{T}\boldsymbol{x}_1 < \boldsymbol{a}_{+}^{T}\boldsymbol{x}_0$ if $\boldsymbol{a}_{-}^{T}\boldsymbol{x}_1 < \boldsymbol{a}_{-}^{T}\boldsymbol{x}_0$. In other words, for any other feasible solution $\boldsymbol{x}_1$, it is impossible that $\boldsymbol{x}_1$ has better performance than $\boldsymbol{x}_0$ in both of the two objectives of maximizing total AAs-to-maximize and minimizing total AAs-to-minimize. If the diet $\boldsymbol{x}_1$ has higher total intake of AAs-to-maximize than the diet $\boldsymbol{x}_0$, then it must have higher total intake of AAs-to-minimize than $\boldsymbol{x}_0$. On the other hand, if the diet $\boldsymbol{x}_1$ has lower total intake of AAs-to-minimize than the diet $\boldsymbol{x}_0$, then it must have lower total intake of AAs-to-maximize than $\boldsymbol{x}_0$. The Pareto surface of the problem is then defined as the set consisting of all Pareto solutions within the feasible region.

To construct the Pareto surface, we applied the ε-Constraint algorithm. Briefly, for a human dietary pattern with defined mathematical form, we first determine the range of total intake of AAs-to-maximize in that dietary pattern using linear programming, $[q, r]$:

$$u = \min \boldsymbol{a}_{+}^{T}\boldsymbol{x}, \text{s.t.}$$

$$\begin{cases} \boldsymbol{x} \geq \mathbf{0} \\ \boldsymbol{l}_n \leq \mathbf{C}\boldsymbol{x} \leq \boldsymbol{u}_n \\ \boldsymbol{l}_f \leq \mathbf{D}\boldsymbol{x} \leq \boldsymbol{u}_f \\ \mathbf{E}\boldsymbol{x} \leq \mathbf{0} \end{cases}$$

$$v = \max \boldsymbol{a}_{+}^{T}\boldsymbol{x}, \text{s.t.}$$

$$\begin{cases} \boldsymbol{x} \geq \mathbf{0} \\ \boldsymbol{l}_n \leq \mathbf{C}\boldsymbol{x} \leq \boldsymbol{u}_n \\ \boldsymbol{l}_f \leq \mathbf{D}\boldsymbol{x} \leq \boldsymbol{u}_f \\ \mathbf{E}\boldsymbol{x} \leq \mathbf{0} \end{cases}$$

Therefore, for any value $s \in [q, r]$, a Pareto solution can be obtained by solving the linear programming problem below:

$$\min \boldsymbol{a}_{-}^{T}\boldsymbol{x}, \text{s.t.}$$

$$\begin{cases} \boldsymbol{x} \geq \boldsymbol{0} \\ \boldsymbol{l}_n \leq \mathbf{C}\boldsymbol{x} \leq \boldsymbol{u}_n \\ \boldsymbol{l}_f \leq \mathbf{D}\boldsymbol{x} \leq \boldsymbol{u}_f \\ \mathbf{E}\boldsymbol{x} \leq \boldsymbol{0} \\ \boldsymbol{a}_+^T\boldsymbol{x} \geq w \end{cases}$$

The Pareto surface of each dietary pattern was constructed by uniformly selecting 100 values of $s \in [q, r]$ and computing the corresponding Pareto solution using the method described above.

5.3 Estimation of deviation from Pareto surface

For each dietary record in the NHANES dataset, we first computed the total daily intake of AAs-to-maximize and AAs-to-minimize in that dietary record: (u, v), and then computed the Euclidean distance between (u, v) and the Pareto surface constructed using the method described in the previous section. Let $\boldsymbol{\Theta} = \{(s_i, t_i)\}_{i=1,2,\cdots,100}$ denote the set consisting of the intake of AAs-to-maximize and AAs-to-minimize in the 100 Pareto solutions computed previously, in which s_i and t_i means the intake of AAs-to-maximize and AAs-to-minimize in the i-th Pareto solution, respectively. We then calculated the deviation from Pareto surface:

$$d(u, v) = \min_{i=1,2,\cdots,100} \|(u, v) - (s_i, t_i)\|_2$$

Since the deviation from the Pareto surface computed this way is largely dependent of the total intake of protein in the dietary record, we adjusted it to the protein intake by fitting a 6-th order polynomial function of protein intake. Let z denote the intake of protein in a dietary record (which has the intake of AAs-to-maximize and AAs-to-minimize being (u, v), we fit the data to the model below:

$$d(u, v) = f(z) = c_0 + c_1 z + c_2 z^2 + c_3 z^3 + c_4 z^4 + c_5 z^5 + c_6 z^6$$

The residue $\hat{d} = d(u, v) - f(z)$ was then defined as the adjusted deviation from Pareto surface, which was used in all downstream analyses related to deviation from Pareto surface (Supplementary Figure 12).

Supplementary References

1 Kaufman, D. E. & Smith, R. L. Direction choice for accelerated convergence in hit-and-

run sampling. *Oper Res* **46**, 84-95, doi:DOI 10.1287/opre.46.1.84 (1998).
2 Andersen, E. D. & Andersen, K. D. in *High Performance Optimization* (eds Hans Frenk, Kees Roos, Tamás Terlaky, & Shuzhong Zhang) 197-232 (Springer US, 2000).
3 Stekhoven, D. J. & Buhlmann, P. MissForest-non-parametric missing value imputation for mixed-type data. *Bioinformatics* **28**, 112-118, doi:10.1093/bioinformatics/btr597 (2012).
4 van Buuren, S. & Groothuis-Oudshoorn, K. mice: Multivariate Imputation by Chained Equations in R. *J Stat Softw* **45**, 1-67 (2011).

Supplementary Figures

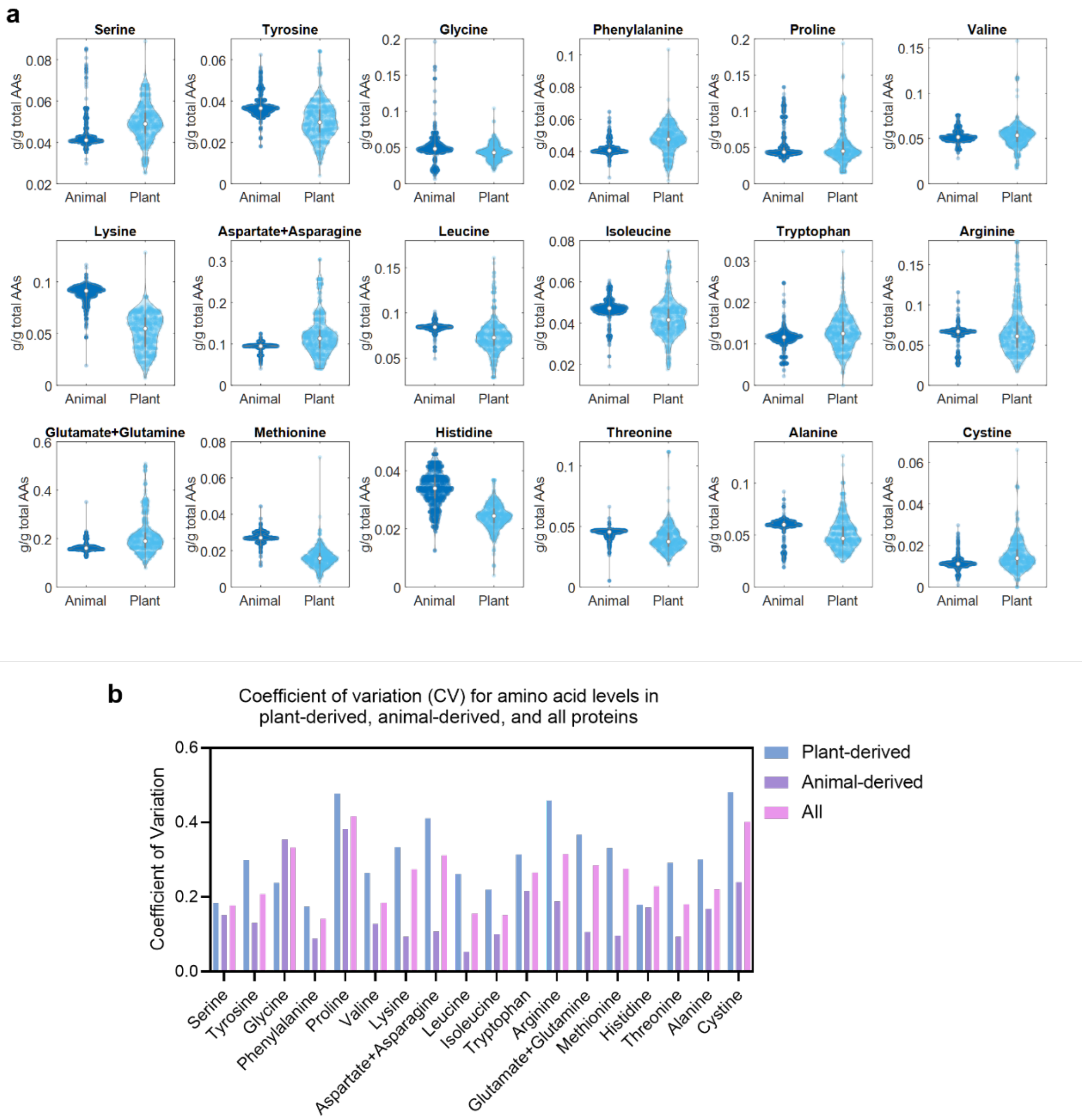

Supplementary Figure 1 (Related to Figure 1) Variability of amino acid profiles in plant-based and animal-based foods.

(a) Violin plots comparing the distributions of amino acid profiles between plant-based and animal-based foods. The circles represent median values, the upper and lower bounds of boxes indicate the range between the 1st and 3rd quartiles, and the whisker ends indicate ranges of data points. n = 1245 for animal-based foods, n = 467 for plant-based foods.

(b) Comparison of coefficient of variation (CV) for amino acid profiles among plant-based, animal-based,

and all foods.

Mediterranean diet

Food	Servings
Cereals	3~6/day
Fish	>=2/week
Fruits	3~6/day
Legumes	>=2/week
White meat	2/week
Processed meat	<=1/week
Sweets	<=2/week
Vegetables	>=6/day
Potatoes	<=3/week
Red meat	<=2/week
Olives/nuts/seeds	1~2/day
Olive oil	>=1/day
Eggs	2~4/week
Dairy	2/day
Others	0/day

Paleo diet

Food	Servings
Game meat/seafood	unlimited
Nuts/seeds	unlimited
Fruits/vegetables	unlimited
Honey	unlimited
Others	0/day

Vegetarian diet

Food	Servings
Meat	0/day
Others	unlimited

Japanese diet

Food	Servings
Cereals	5~7/day
Fruits	2/day
Vegetables	5~6/day
Dairy	2/day
Proteins	3~5/day
Others	0/day

American diet

Food	Consumption (+/-20%)
Meat	381g/day
Sweets	166g/day
Fats/oils	114g/day
Grains	291g/day
Starchy roots	164g/day
Vegetables	310g/day
Fruits	266g/day
Eggs	38g/day
Milk	704g/day
Legumes	9g/day
Alcoholic beverages	258g/day
Others	28g/day
**Nutrient**	**Intake (+/-20%)**
Calories	3641 kcal/day

Ketogenic diet

Nutrient	% kcal
Fat	>=70%
Carbohydrate	<=5%

USDA-recommended diet

Nutrient	Intake
Calories	1800~2200kcal/day
Protein	>=46g/day, 10~35%kcal
Carbohydrate	>=130g/day, 45~65%kcal
Dietary fiber	>=28g/day
Sugar	<=10%kcal
Fat	20~35%kcal
Saturated fat	<=10%kcal
Linoleic acid	>=12g/day
Linolenic acid	>=1.1g/day
Calcium	>=1g/day
Iron	>=18mg/day
Magnesium	>=310mg/day
Phosphorus	>=700mg/day
Potassium	>=4.7g/day
Sodium	<=2.3g/day
Zinc	>=8mg/day
Copper	>=0.9mg/day
Manganese	>=1.8mg/day
Selenium	>=55μg/day
Vitamin A	>=700mg/day
Vitamin E	>=15mg/day
Vitamin D	>=600IU/day
Vitamin C	>=75mg/day

Atkins diet

Nutrient	Intake
Carbohydrate	<=20g/day

USDA-recommended diet (continued)

Nutrient	Intake
Thiamin	>=1.1mg/day
Riboflavin	>=1.1mg/day
Niacin	>=14mg/day
Vitamin B6	>=1.3mg/day
Vitamin B12	>=2.4μg/day
Choline	>=425mg/day
Vitamin K	>=90μg/day
Folate	>=400μg/day

DASH diet

Food	Servings
Grains	6~8/day
Vegetables	4~5/day
Fruits	4~5/day
Low-fat dairy	2~3/day
Lean meat	<=6/day
Nuts/seeds/legumes	4~5/week
Fats/oils	2~3/day
Sweets	<=5/week
Others	0/day
**Nutrient**	**Intake**
Sodium	<=2.3g/day

Plant-based diet

Food	Servings
Meat	0/day
Dairy	0/day
Eggs	0/day
Others	unlimited

Supplementary Figure 2 (Related to Figure 2). Definition of human dietary patterns. Definitions of the 10 dietary patterns considered in this study, including Mediterranean diet, Japanese diet, American diet, Paleo diet, vegetarian diet, plant-based diet, Atkins diet, ketogenic diet, Diet Approaches to Stop Hypertension (DASH diet), and the United States of America Department of Agriculture (USDA)-recommended diet, are shown.

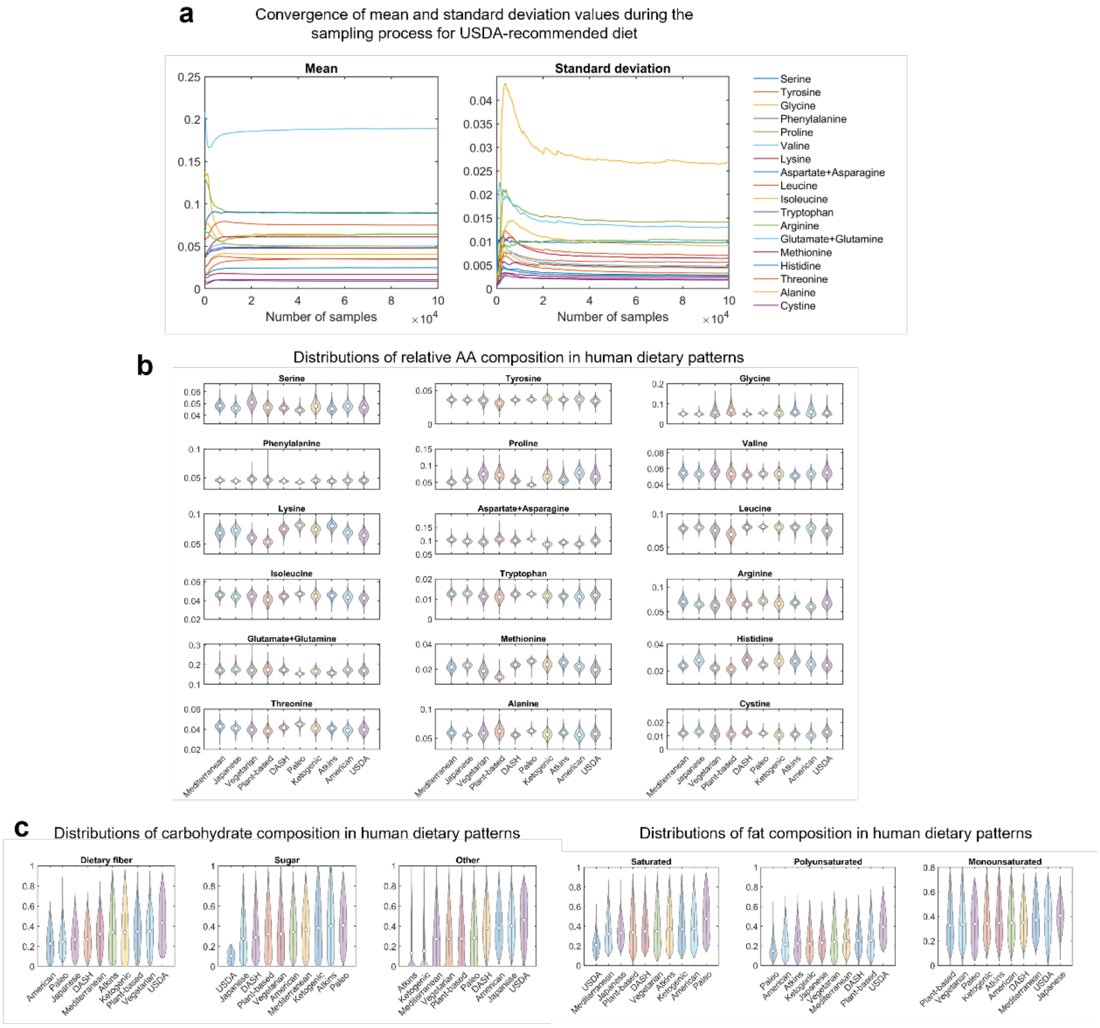

Supplementary Figure 3 (Related to Figure 2). Amino acids, carbohydrates, and fats in human dietary patterns.

(a) Convergence of mean and standard deviation values during the sampling of diets under the dietary pattern "the United States of America Department of Agriculture (USDA)-recommended diet".

(b) Distributions of relative amino acid composition in human dietary patterns.

(c) Distributions of relative carbohydrate composition and relative fat composition in human dietary patterns.

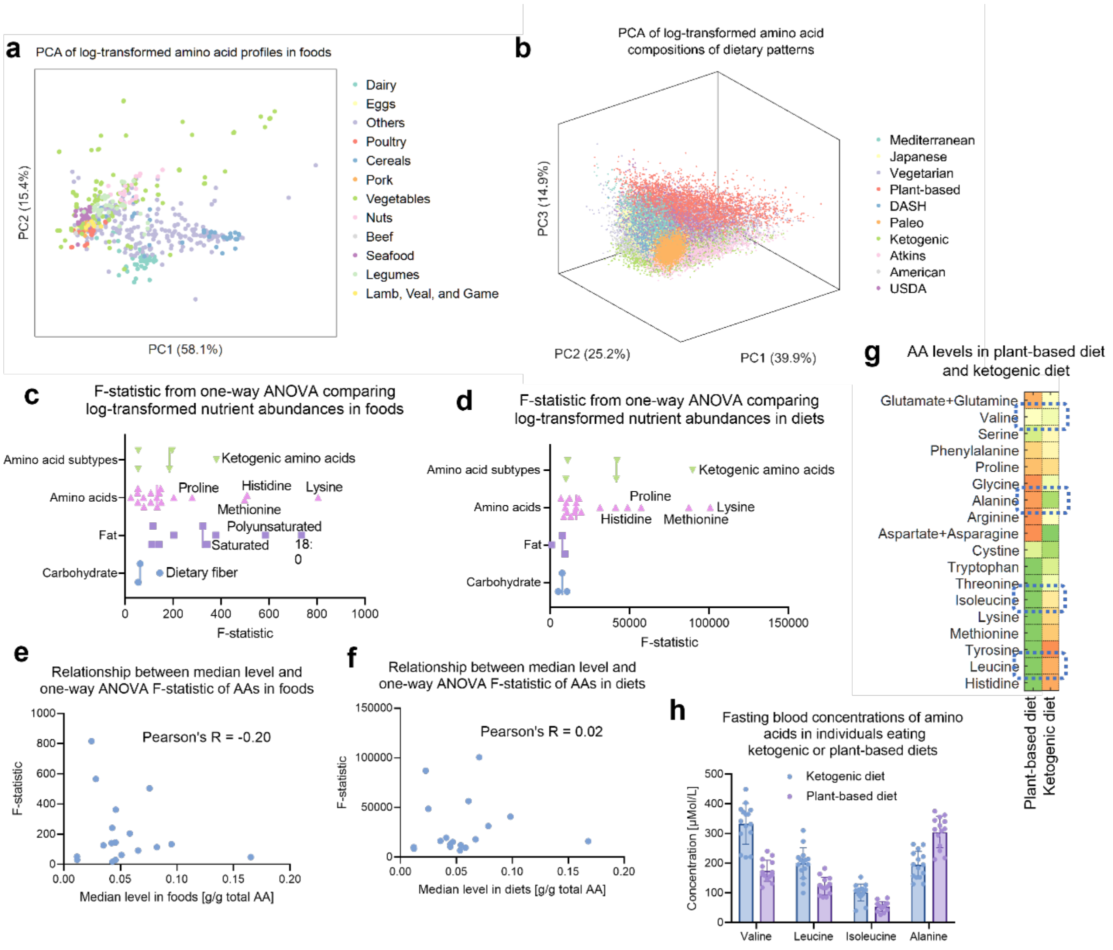

Supplementary Figure 4 (Related to Figure 2). Amino acids in human foods and dietary patterns.

(a) Principal components analysis (PCA) of log-transformed amino acid compositions in human foods.

(b) Principal components analysis (PCA) of log-transformed amino acid compositions in human dietary patterns.

(c) F-statistic from one-way analysis of variance (ANOVA) comparing log-transformed nutrient abundances in human foods.

(d) F-statistic from one-way analysis of variance (ANOVA) comparing log-transformed nutrient abundances in human dietary patterns.

(e) Scatter plot comparing median level and F-statistic of amino acids in human foods.

(f) Scatter plot comparing median level and F-statistic of amino acids in human dietary patterns.

(g) Amino acid levels in plant-based and ketogenic diets estimated by our computational method.

(h) Fasting blood amino acid concentrations in human individuals eating plant-based or ketogenic diet.

The error bars indicate standard deviation. n = 15 for ketogenic diet, n = 14 for plant-based diet.

a

Three variations of the Atkins diet

Variation 1: the original Atkins diet

Nutrient	Intake
Carbohydrate	<=20g/day

Variation 2: Atkins diet at the 'ongoing weight loss' phase

Nutrient	Intake
Carbohydrate	<=50g/day

Variation 3: Atkins diet with limited consumption of red meat

Nutrient	Intake
Carbohydrate	<=20g/day
Red meat	0g/day

b

Three variations of the Mediterranean diet

Variation 1: the pyramid for Mediterranean diet

Food	Servings
Cereals	3~6/day
Fish	>=2/week
Fruits	3~6/day
Legumes	>=2/week
White meat	2/week
Processed meat	<=1/week
Sweets	<=2/week
Vegetables	>=6/day
Potatoes	<=3/week
Red meat	<=2/week
Olives/nuts/seeds	1~2/day
Olive oil	>=1/day
Eggs	2~4/week
Dairy	2/day
Others	0/day

Variation 2: the traditional Mediterranean diet

Food	Servings
Whole-grain cereals	8/day
Fish	5~6/week
Fruits	3/day
Poultry	4/week
Sweets	<=3/week
Vegetables	6/day
Potatoes	3/week
Red meat	<=4/month
Olives/nuts/seeds	3~4/week
Olive oil	>=1/day
Eggs	3/week
Dairy	2/day
Others	0/day

Variation 3: Diet with highest Mediterranean diet score (MDS)

Dietary variable	Range
Vegetables	>=499.6g/day
Legumes	>=6.7g/day
Fruits and nuts	>=356.3g/day
Cereals	>=139.7g/day
Fish	>=18.8g/day
Meat	<=89.8g/day
Dairy	<=191.1g/day
Alcohol	5~25g/day
Monounsaturated-to-saturated ratio	>=1.7

c

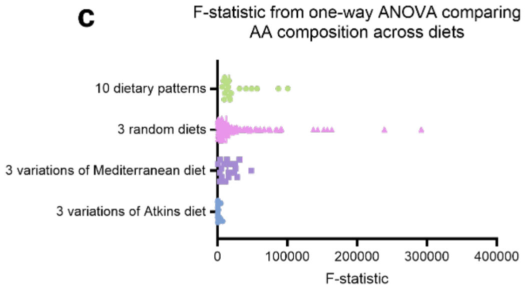

d

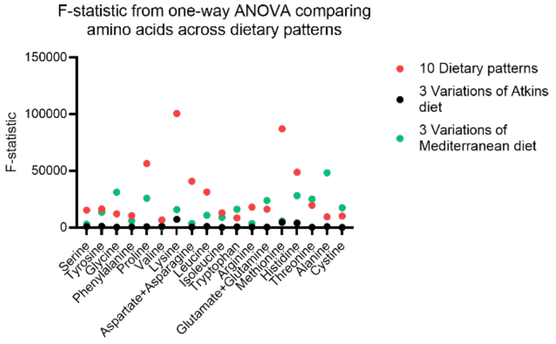

Supplementary Figure 5 (Related to Figure 2). Amino acid signatures of the variations of Atkins diet and Mediterranean diet.

(a) Definitions of the three variations of Atkins diet.

(b) Definitions of the three variations of Mediterranean diet.

(c) Swarm plots showing the distributions of F-statistic from one-way analysis of variance (ANOVA) comparing amino acid levels across the original 10 dietary patterns, three random dietary patterns from the original 10 dietary patterns, three variations of Mediterranean diet, and three variations of Atkins

diet.

(d) Scatter plots showing the F-statistic values from one-way analysis of variance (ANOVA) comparing amino acid levels across the original 10 dietary patterns, three variations of Mediterranean diet, and three variations of Atkins diet.

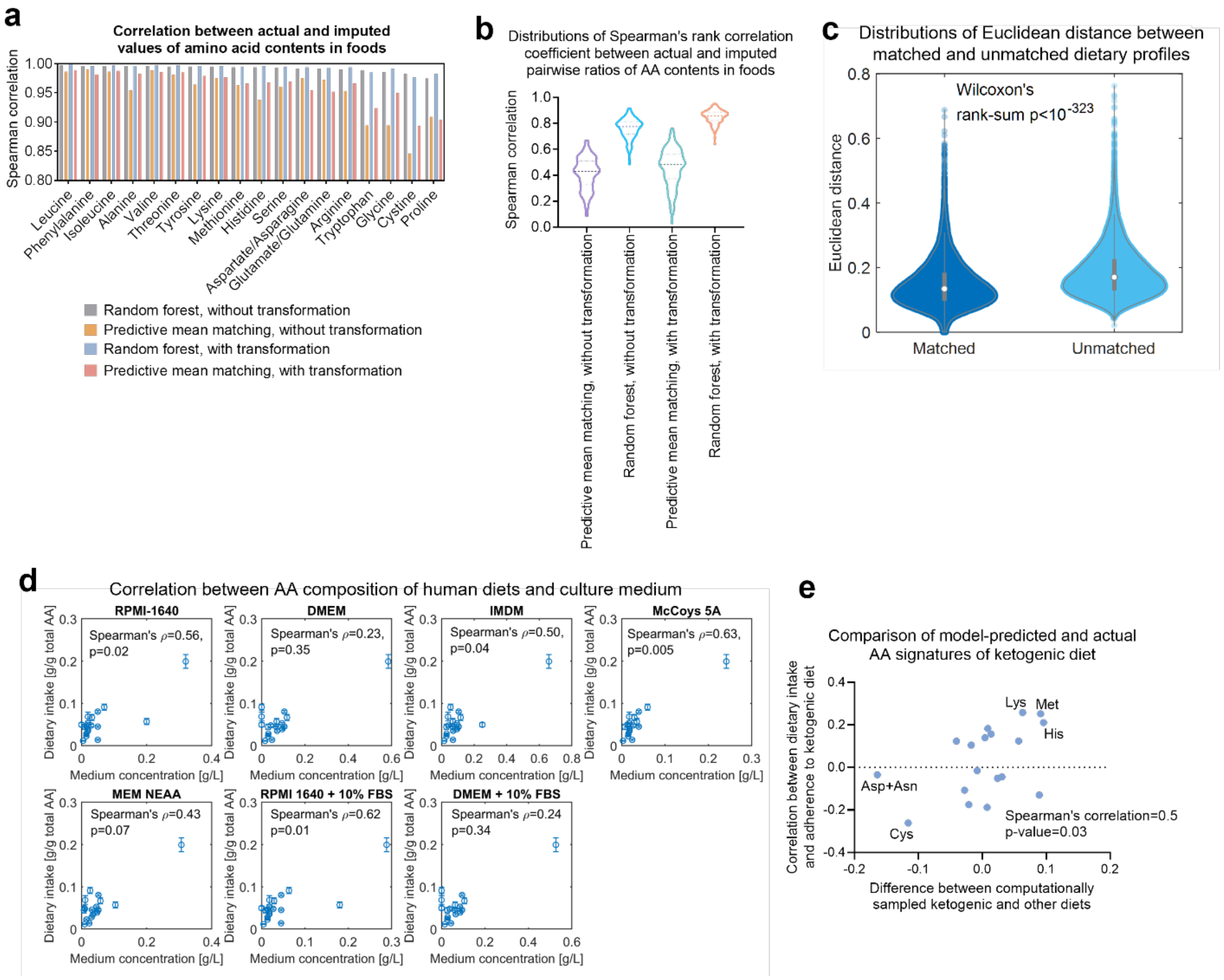

Supplementary Figure 6 (Related to Figure 3). Imputation and validation of amino acid intake profiles in human dietary records.

(a) Spearman's rank correlation coefficients between actual and imputed values of amino acid abundances in foods using different algorithms for data imputation.

(b) Distributions of Spearman's rank correlation coefficients between actual and imputed pairwise ratios of amino acid abundances in foods using different algorithms for data imputation.

(c) Distributions of Euclidean distance between matched (two 24-hour recalls of the same person) and unmatched (two 24-hour recalls from different individuals) dietary intake profiles. The circles represent median values, the upper and lower bounds of boxes indicate the range between the 1st and 3rd quartiles, and the whisker ends indicate ranges of data points. The p-value was calculated by two-sided Wilcoxon's rank-sum test. Sample size n = 30921.

(d) Comparison between concentrations of amino acids in culture mediums and imputed human dietary amino acid intakes. The error bars indicate standard deviation of the data. The p-values were calculated by two-sided Spearman's rank correlation test. Sample size n = 30899 for dietary intake profiles, n = 1 for culture medium.

(e) Comparison of model-predicted and actual amino acid signatures of the ketogenic diet. The p-value was calculated by two-sided Spearman's rank correlation test.

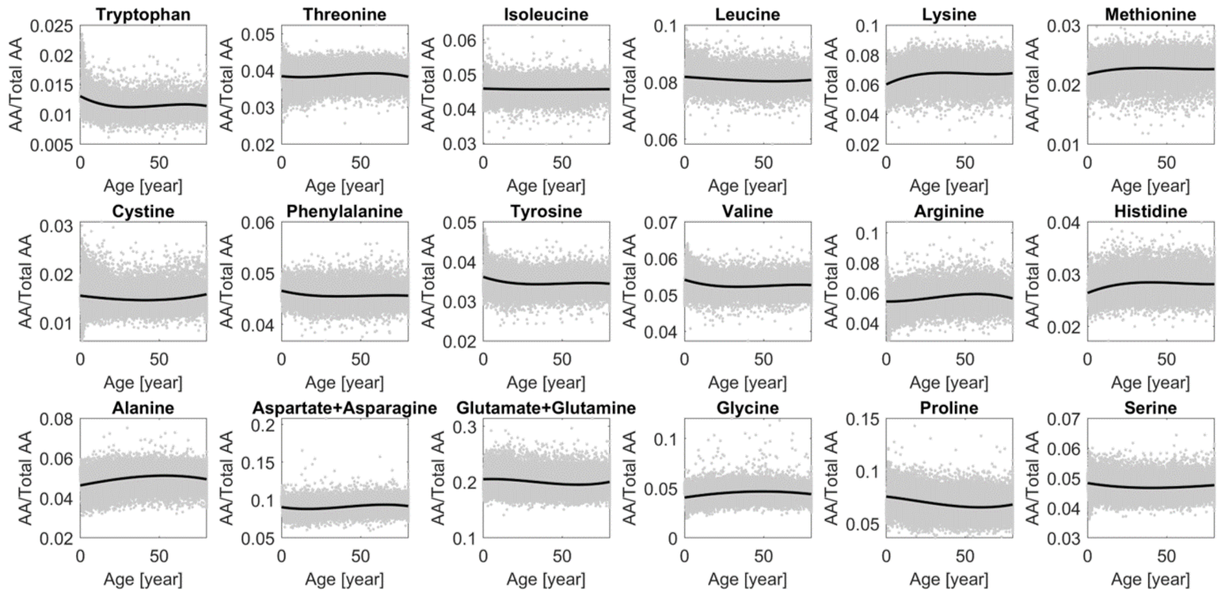

Supplementary Figure 7 (Related to Figure 3). Relationship between dietary amino acid intake and age in U.S. citizens. Scatter plots indicate the raw data in the NHANES datasets. Solid curves indicate optimal fit of the data points using a polynomial function with order three.

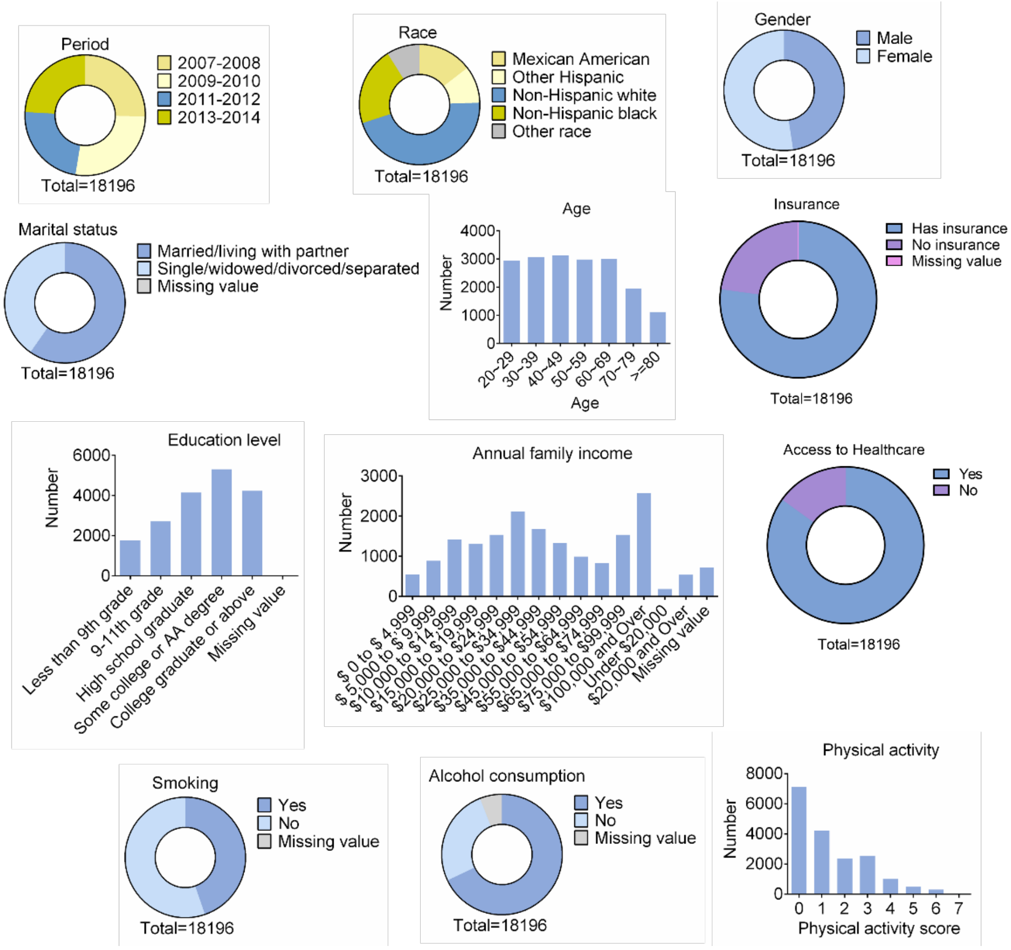

Supplementary Figure 8 (Related to Figure 4). Demographic characteristics of the population.

Distribution of the batch, race, gender, marital status, age, insurance coverage, education level, annual family income, access to healthcare, smoking history, alcohol consumption, and physical activity are shown.

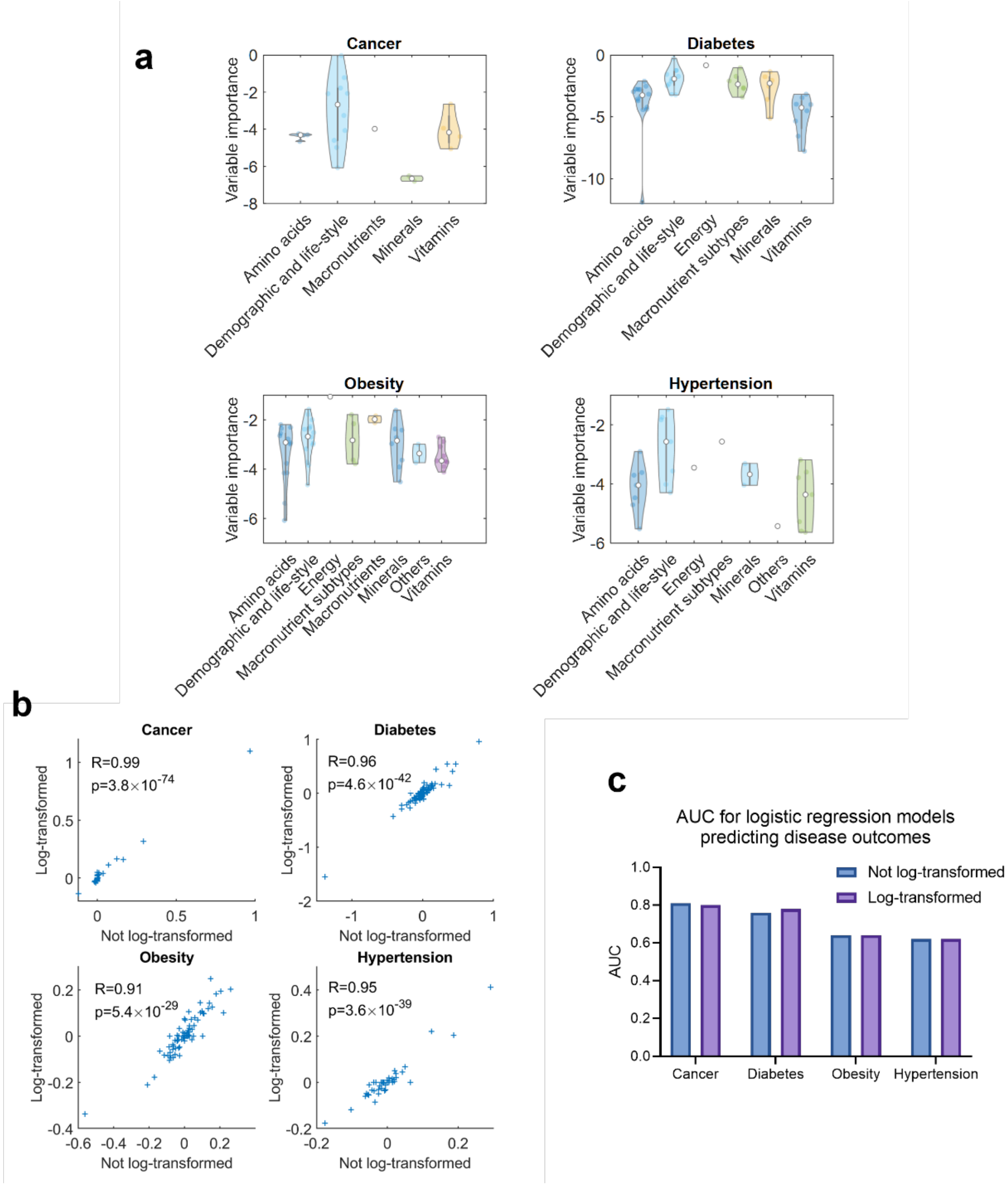

Supplementary Figure 9 (Related to Figure 4). Machine learning model predicting disease prevalence from dietary variables.

(a) Violin plots comparing the distribution of variable importance in different categories of variables. The circles represent median values, the upper and lower bounds of boxes indicate the range between the 1st and 3rd quartiles, and the whisker ends indicate ranges of data points.

(b) Scatter plots comparing the standardized regression coefficients between the model using original non-transformed variables and the model using log-transformed variables.

(c) Bar plots comparing the AUC values for the models predicting disease outcomes from dietary variables using the original variables or log-transformed variables.

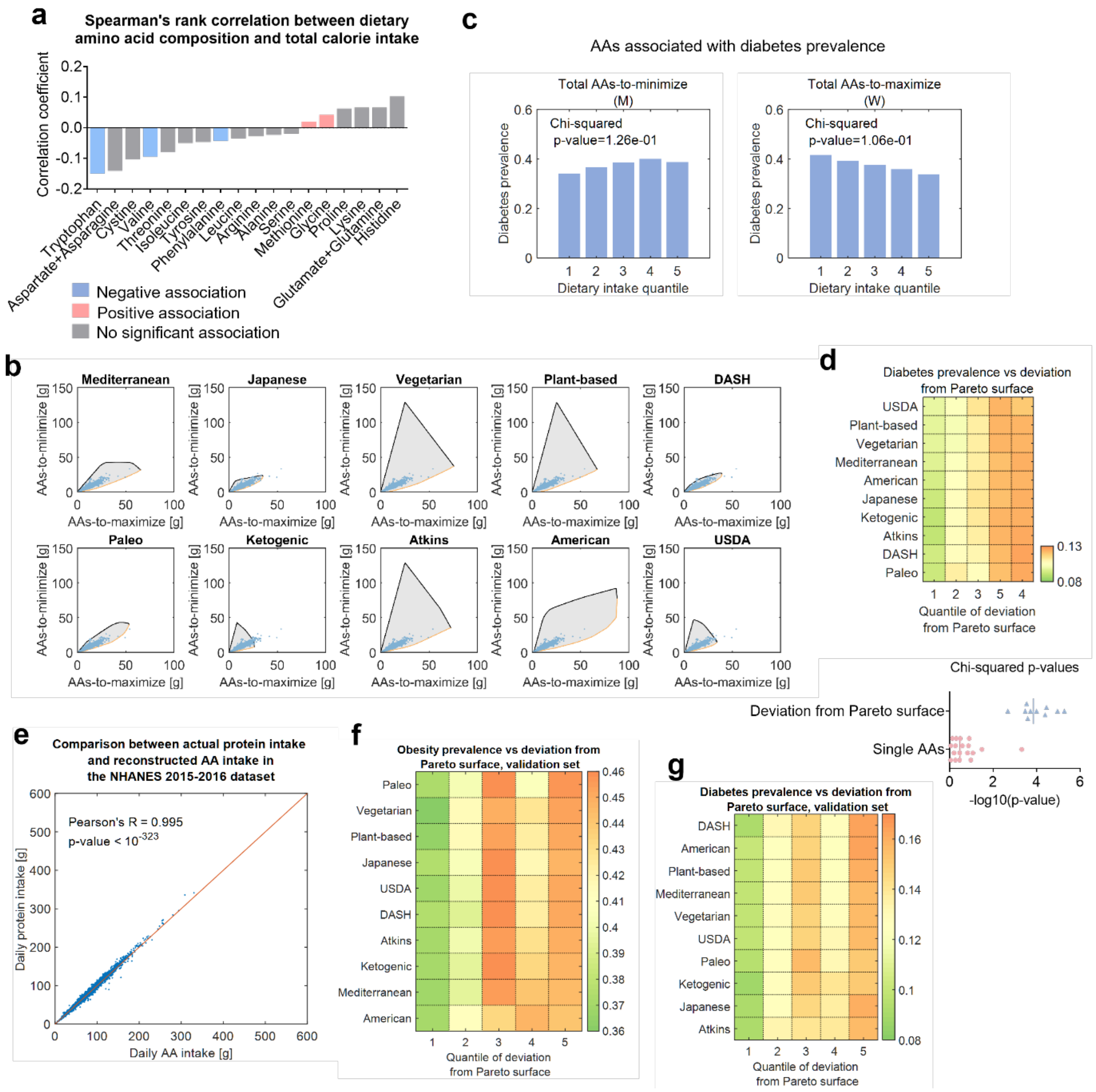

Supplementary Figure 10 (Related to Figure 5). Association between dietary amino acid intake and obesity.

(a) Spearman's rank correlation coefficients between dietary intake of amino acids and total calorie intake.

(b) Ranges of intake of total amino-acids-to-maximize and amino-acids-to-minimize in the 10 human dietary patterns (grey shaded regions), the Pareto surface (orange bold curve) corresponding to the two guidelines, i.e. maximizing total amino-acids-to-maximize, and minimizing total amino-acids-to-minimize, and actual intake of total amino-acids-to-maximize and total-amino-acids-to-minimize in

human dietary records in the NHANES datasets.

(c) Prevalence of diabetes in human subjects with different levels of intake of the amino acids positively or negatively associated with diabetes. The p-values were calculated by two-sided chi-squared test.

(d) Associations between the diabetes prevalence and deviation of dietary intake profiles from the Pareto surface. Chi-squared p-values were computed to assess the significance levels of the associations The p-values were calculated by two-sided chi-squared test. Sample size n = 18 for single amino acids, n = 10 for deviation from Pareto surface.

(e) Scatter plot comparing the reconstructed total dietary amino acid intake and dietary protein intake in the NHANES 2015-2016 dataset, which was used as an external validation set. The p-value was calculated by two-sided Pearson's correlation test.

(f) Heatmap showing the association between the obesity prevalence and deviation of dietary intake profiles from the Pareto surface in the validation set. n = 4172 dietary intake profiles.

(g) Heatmap showing the association between the diabetes prevalence and deviation of dietary intake profiles from the Pareto surface in the validation set. n = 4172 dietary intake profiles.

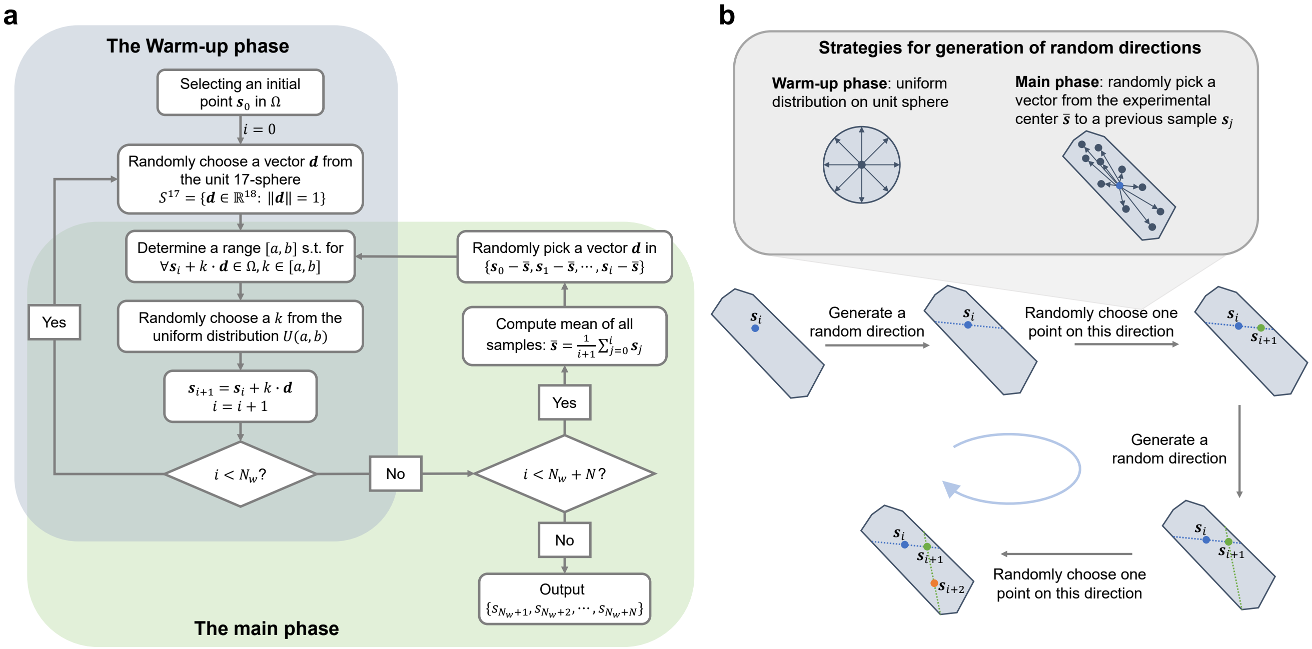

Supplementary Figure 11. Workflow of the hit-and-run sampling algorithm for random sampling of diets under a specific dietary pattern.

(a) Workflow of the hit-and-run algorithm for uniform sampling of the feasible region for the amino acid abundance vector $\boldsymbol{s} = \boldsymbol{A}\boldsymbol{x}$.

(b) Illustration of each iterative step and strategies for generation of random directions in the hit-and-run algorithm.

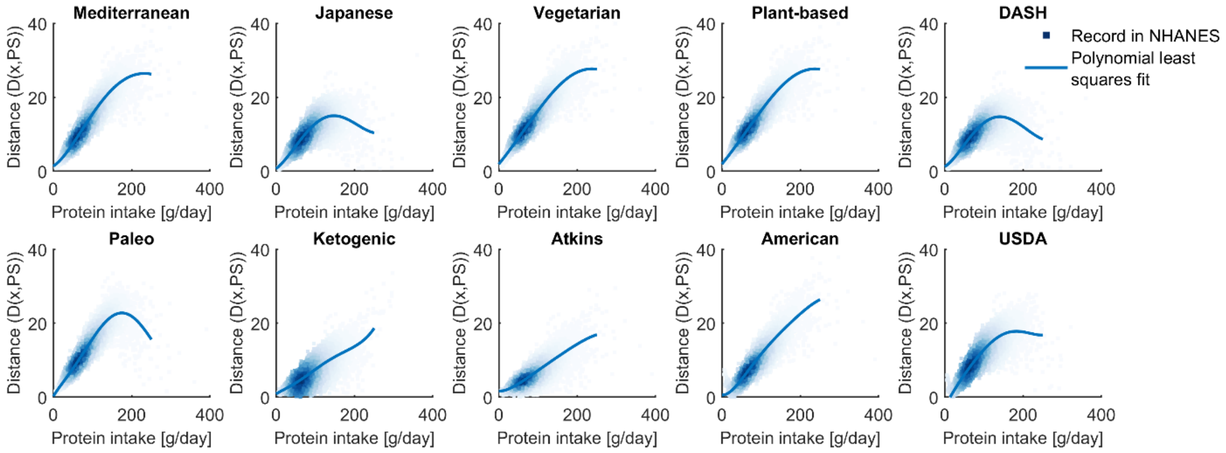

Supplementary Figure 12. Adjustment of the deviation from Pareto surface to total protein intake. A polynomial function with order six was used to fit the relationship between dietary protein intake and the deviation from Pareto surface. Density scatter plots indicate distribution of data points in the NHANES datasets. Solid curves indicate the optimal fit.
