# Supplementary material for: Amino acid variability, tradeoffs and optimality in human diet
Source: Nat Commun. 2022 Nov 5;13:6683. doi: 10.1038/s41467-022-34486-0 (PMC9637229; doi:10.1038/s41467-022-34486-0)
Supplement: Supplementary file 3 — Reporting Summary [file 41467_2022_34486_MOESM3_ESM.pdf]

nature portfolio

Corresponding author(s): Jason W Locasale

Last updated by author(s): Oct 15, 2022

Reporting Summary

Nature Portfolio wishes to improve the reproducibility of the work that we publish. This form provides structure for consistency and transparency in reporting. For further information on Nature Portfolio policies, see our Editorial Policies and the Editorial Policy Checklist.

Statistics

For all statistical analyses, confirm that the following items are present in the figure legend, table legend, main text, or Methods section.

n/a	Confirmed	
☐	☒	The exact sample size (n) for each experimental group/condition, given as a discrete number and unit of measurement
☐	☒	A statement on whether measurements were taken from distinct samples or whether the same sample was measured repeatedly
☐	☒	The statistical test(s) used AND whether they are one- or two-sided<br>*Only common tests should be described solely by name; describe more complex techniques in the Methods section.*
☐	☒	A description of all covariates tested
☐	☒	A description of any assumptions or corrections, such as tests of normality and adjustment for multiple comparisons
☐	☒	A full description of the statistical parameters including central tendency (e.g. means) or other basic estimates (e.g. regression coefficient) AND variation (e.g. standard deviation) or associated estimates of uncertainty (e.g. confidence intervals)
☐	☒	For null hypothesis testing, the test statistic (e.g. F, t, r) with confidence intervals, effect sizes, degrees of freedom and P value noted<br>*Give P values as exact values whenever suitable.*
☒	☐	For Bayesian analysis, information on the choice of priors and Markov chain Monte Carlo settings
☒	☐	For hierarchical and complex designs, identification of the appropriate level for tests and full reporting of outcomes
☐	☒	Estimates of effect sizes (e.g. Cohen's d, Pearson's r), indicating how they were calculated

Our web collection on statistics for biologists contains articles on many of the points above.

Software and code

Policy information about availability of computer code

Data collection	No software was used for data collection because all datasets used in this study were downloaded from public databases
Data analysis	All code and scripts used or generated in this study are available at the GitHub page of Ziwei Dai: https://github.com/ziweidai/AA_human_diet. The software MATLAB and R are required to execute the custom scripts.

For manuscripts utilizing custom algorithms or software that are central to the research but not yet described in published literature, software must be made available to editors and reviewers. We strongly encourage code deposition in a community repository (e.g. GitHub). See the Nature Portfolio guidelines for submitting code & software for further information.

Data

Policy information about availability of data

All manuscripts must include a data availability statement. This statement should provide the following information, where applicable:

- Accession codes, unique identifiers, or web links for publicly available datasets
- A description of any restrictions on data availability
- For clinical datasets or third party data, please ensure that the statement adheres to our policy

Datasets used in this study were obtained from the public databases USDA SR, FNDDS, and NHANES. Briefly, Microsoft Access database files for USDA National Nutrient Database for Standard Reference (SR) and the USDA Food and Nutrient Database for Dietary Studies (FNDDS) were downloaded from the website for USDA Agricultural Research Service: https://www.ars.usda.gov/northeast-area/beltsville-md-bhnrc/beltsville-human-nutrition-research-center/methods-and-application-of-food-composition-laboratory/mafcl-site-pages/sr17-sr28/ (SR), and https://www.ars.usda.gov/northeast-area/beltsville-md-bhnrc/beltsville-human-nutrition-

research-center/food-surveys-research-group/docs/fndds-download-databases/ (FNDDS). SAS (.xpt) files for NHANES 2007-2008, 2009-2010, 2011-2012, 2013-2014 datasets, including demographics data, dietary data, examination data, laboratory data and questionnaire data were retrieved from the web page for NHANES: https://wwwn.cdc.gov/nchs/nhanes/Default.aspx. All datasets used or generated in this study are also available at the GitHub page of Ziwei Dai: https://github.com/ziweidai/AA_human_diet.

Human research participants

Policy information about studies involving human research participants and Sex and Gender in Research.

Reporting on sex and gender	Information about gender is provided in Figure S8
Population characteristics	Information about population characteristics is provided in Figure S8
Recruitment	The analyses were done with data in public database
Ethics oversight	N/A

Note that full information on the approval of the study protocol must also be provided in the manuscript.

Field-specific reporting

Please select the one below that is the best fit for your research. If you are not sure, read the appropriate sections before making your selection.

- [x] Life sciences
- [ ] Behavioural & social sciences
- [ ] Ecological, evolutionary & environmental sciences

For a reference copy of the document with all sections, see nature.com/documents/nr-reporting-summary-flat.pdf

Life sciences study design

All studies must disclose on these points even when the disclosure is negative.

Sample size	In the analysis of relationship between dietary amino acids and human diseases, the sample size (i.e. number of individuals in the analysis of human dietary intake profiles) was determined based on the total number of entries in the NHANES datasets. In the analysis of amino acid landscape in human dietary patterns, the sample size (i.e. number of random diets sampled under each dietary pattern) was determined based on the criteria of convergence for MCMC sampling algorithm.
Data exclusions	Non-adult individuals (i.e. age <= 20 years old) were not included in the analysis. Individuals with dietary intake of any nutrient higher than three times of the 99th percentile of the intake of that nutrient among the population were considered outliers and not included in the following analysis.
Replication	The computational analyses performed in this study were repeated once because the algorithms for data analysis, except for the random sampling of diets under a certain dietary pattern, are deterministic and do not require random seeds. Random sampling of diets under each dietary pattern was repeated for 50,000 times to provide sufficiently large sample size.
Randomization	Regarding the analysis of relationship between dietary amino acids and human diseases, randomization of samples was done by assigning a survey weight to each individual for the entire dataset to be representative of the population of the U.S. The survey weights were included in the original NHANES datasets and considered in training the machine learning model.
Blinding	Not relevant because this study includes only computational analysis

Reporting for specific materials, systems and methods

We require information from authors about some types of materials, experimental systems and methods used in many studies. Here, indicate whether each material, system or method listed is relevant to your study. If you are not sure if a list item applies to your research, read the appropriate section before selecting a response.

Materials & experimental systems

n/a	Involved in the study	
☒	☐	Antibodies
☒	☐	Eukaryotic cell lines
☒	☐	Palaeontology and archaeology
☒	☐	Animals and other organisms
☒	☐	Clinical data
☒	☐	Dual use research of concern

Methods

n/a	Involved in the study	
☒	☐	ChIP-seq
☒	☐	Flow cytometry
☒	☐	MRI-based neuroimaging
